# Effects of ginsenoside Rb1 on spinal cord ischemia-reperfusion injury in rats

**DOI:** 10.1186/s13018-019-1299-2

**Published:** 2019-08-14

**Authors:** Jin-tao Ye, Feng-tao Li, Sheng-li Huang, Jian-li Xue, Yirixiati Aihaiti, Hao Wu, Ruo-xi Liu, Bin Cheng

**Affiliations:** 1 Department of Orthopaedics, Xi’an Jiaotong University Second Affiliated Hospital, No. 157 Xiwu Road, Xi’an, 710004 Shaanxi Province People’s Republic of China; 2 Department of Joint Surgery, Xi’an Hong Hui Hospital, Xi’an Jiaotong University Health Science Center, No. 55 Youyidong Road, Xi’an, 710000 Shaanxi Province China

**Keywords:** Spinal cord Injury, Ischemia-reperfusion Injury, Oxidative stress, Ginsenoside Rb1, Survivin protein

## Abstract

**Background:**

The aim of this study was to evaluate the effects of different doses of ginsenoside Rb1 (GRb1) pretreatment on spinal cord ischemia-reperfusion (SCII) in rats and explore the potential mechanisms about the expression of survivin protein after the intervention.

**Methods:**

A total of 90 healthy adult Sprague-Dawley (SD) rats were randomly divided into six groups: sham-operated (*n* = 15), SCII model (*n* = 15), and GRb1-treated groups (*n* = 60). The GRb1-treated group was divided into four subgroups: 10 mg/kg, 20 mg/kg, 40 mg/kg, and 80 mg/kg (*n* = 15). The corresponding dose of GRb1 was injected intraperitoneally 30 min before operation and every day after operation. Forty-eight hours after model establishment, the neurological function of hind limbs was measured with Basso, Beattie, and Bresnahan (BBB) scale. The superoxide dismutase (SOD) and malondialdehyde (MDA) levels in serum and spinal cord tissue were detected respectively. The expression of survivin protein was observed by immunofluorescence staining. HE and TUNEL staining were used to observe neural cell injury and apoptosis, respectively, in the spinal cord of rats with SCII.

**Results:**

The intervention of different doses of GRb1 could increase SOD activity and decrease MDA content in serum and spinal cord tissue, increase survivin protein expression, and decrease neuronal apoptosis. It was dose-dependent, but there was no significant change between 40 mg/kg and 80 mg/kg.

**Conclusions:**

GRb1 could reduce the cell apoptosis induced by SCII through inhibiting oxidative stress. It can also inhibit apoptosis by promoting the expression of Survivin protein. Ginsenoside Rb1 had a dose-dependent protective effect on SCII in the dose range of 10 mg/kg–40 mg/kg.

## Introduction

Spinal cord ischemia-reperfusion injury (SCII) refers to the phenomenon that the nerve function could not be recovered after a period of ischemia and blood reperfusion, on the contrary, the nerve function was damaged and aggravated [1, 2]. SCII could occur in a variety of conditions, such as spinal trauma, vascular surgery, and the like. Spinal cord tissue belonged to nerve tissue. So far, most studies still believe that nerve tissue ischemia-reperfusion injury was a disease without effective treatment. At present, the general treatment method was that once SCII is found, immediate treatment with large doses of methylprednisolone and neurotrophic drugs could promote the recovery of damaged spinal cord function and prevent further aggravation of injury [3, 4].

**Fig. 1:**
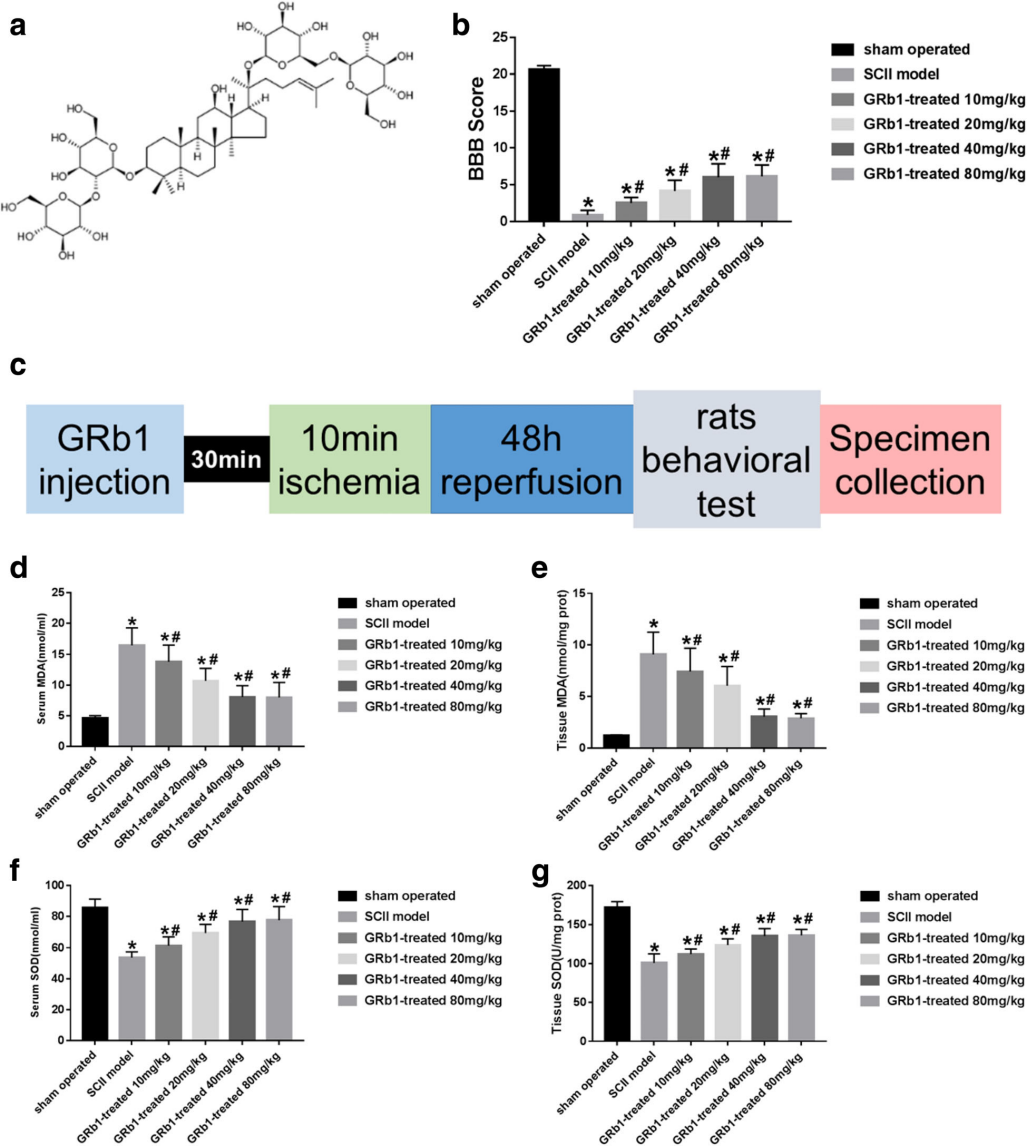
**a** Chemical structure of GRb1. **b** Neurological functional assessment measured by BBB. In general, during the whole experimental period, GRb1-treated group showed significantly better function in comparison with the SCII group. In the sham group, neurological functions of rats were basically no influence. **c** Timeline of GRb1 injection (IP) during the ischemia-reperfusion procedure. **d**–**e** Oxidant stress marker parameter results of MDA in serum and spinal cord tissue. **f**–**g** Oxidant stress marker parameter results of SOD in serum and spinal cord tissue. Although the SOD values of each subgroup in the drug group were higher than the sham group, the values of each time node were lower than those in the SCII group. The SOD values of each subgroup in the drug group were lower than the sham group, but the values of all time nodes were higher than those in the SCII group. (*n* = 15 in each group, *P < 0.05, compared with the sham group; #*P* < 0.05, GRb1 vs. SCII group)

GRb1 belonged to panaxadiol (Fig. 1a), which had anti-oxidation, anti-apoptotic and anti-tumor effects [5–8]. GRb1 had protective effects on ischemic brain injury, and its effect was similar to that of neurotrophic factor [9]. It acts as a free radical scavenger when free radicals are produced in large quantities after cerebral ischemia. Previous studies have shown that GRb1 can significantly alleviate ischemia-reperfusion injury of kidney and brain, but there are few reports on SCII [10]. This study was to explore the relationship between survivin protein and spinal cord ischemia-reperfusion injury, and the possible mechanism of different doses of GRb1 in the treatment of spinal cord ischemia-reperfusion injury.

## Materials and methods

### Animals

Ninety adult male Sprague–Dawley rats (weighing 200 to 230 g) were purchased from the Experimental Animals Center of Xi’an Jiao-tong University. All animals were housed in polypropylene cages (at room temperature of 22 ± 3 °C, relative humidity of 50 ± 15%, and 12-h light/dark cycle) and allowed free access to standard rodent chow and water. None of the animals had any neurological abnormality before anesthesia and surgery. All animal experiment procedures were conducted in accordance with the policies of our university and NIH Guidelines for the Care and Use of Laboratory Animals. Xi’an Jiaotong University Approval for Research Involving Animal No. XJTULAC2018-454.

### Chemicals and reagents

GRg1 powder with high purity (> 98% of the total weight) was purchased from Shanghai Winherb Medical Science Co. Ltd. (Shanghai, China). GRg1 solution was prepared and injected at a concentration of 10 mg/ml.

Mouse monoclonal survivin antibody was obtained from Santa Cruz (CA, 95060, USA). Terminal dUTP nick-end labeling (TUNEL) assay was purchased from Nanjing Jiancheng Bioengineering Institute (Nanjing, China). Superoxide dismutase activity test kit and Malondialdehyde test kit were obtained from Nanjing Jiancheng Bioengineering Institute (Nanjing, China).

### Experiment protocols

Ninety male SD rats were randomly divided into 6 groups with 15 rats in each group. There were a sham group, an SCII model, and four treatment groups. (*n* = 15 per group). In SCII model groups, rats underwent spinal cord ischemia-reperfusion and injected with an equal volume of saline. In GRb1-treated groups, rats received GRb1 administration 30 min before ischemia-reperfusion and the same do as before every day until being sacrificed. GRb1-treated group was divided into four subgroups: 10 mg/kg, 20 mg/kg, 40 mg/kg, and 80 mg/kg.

### SCII model

SCII was induced as described previously by Hwang with slight modifications [11]. In brief, after overnight fasting with unrestricted access to water, the animals were anesthetized intraperitoneally with chloral hydrate (intraperitoneal injection (i.p.), 400 mg/kg) and placed in the supine position. The left carotid artery was exposed and cannulated with a catheter connecting to an external blood reservoir to monitor the proximal arterial pressure and maintain it at 80 mmHg during the aortic occlusion. The tail artery was cannulated with a polyethylene catheter for intraarterial infusion of heparin and the monitoring of distal arterial pressure. To induce spinal cord ischemia, the left femoral artery was exposed and a balloon-tipped 2F catheter (Edwards Life Science, Shanghai, China) was inserted into the descending thoracic aorta (10–12 cm from the site of insertion). The catheter balloon was inflated with 0.05 ml saline and maintained for 10 min. After ischemia, the balloon was deflated and the drained blood was reinfused slowly through the carotid artery catheter. Then, all catheters were removed and the wounds were closed (Fig. 1c). The rats were returned to their cages and allowed to recover. Protamine sulfate (4 mg, i.p.) was given to neutralize excessive heparin, and the bladder content was expelled via manual compression as required.

### Rats behavioral test

After SCII, behavioral test for the motor function was examined at 48 h using the Basso, Beattie, and Bresnahan motor rating scale [12]. The judgers were blinded to experimental conditions and are familiar with the BBB score.

### Specimen collection

Rats were sacrificed after rats behavioral test for each group. After chloral hydrate (400 mg/kg, i.p.) anesthesia finished, 3 ml of blood was collected from the heart. After standing at indoor temperature for 2 h, the supernatant was centrifuged at 1000 rpm for 10 min to obtain serum, which will be stored in a refrigerator at − 20 °C for testing. Some rats immediately removed the lumbar spinal cord tissue about 1 cm in length for SOD and MDA. Tissue specimen were washed by frozen saline and immediately prepared as homogenates (1:10) then centrifuged (14,000 r/min, 4 °C, 15 min); supernatant layer was derived and immediately frozen in liquid nitrogen and stored at − 70 °C until further processing. The rest of the rats opened left aortic ascending aorta, and the right atrium was exposed at the same time. After rapid lavage with 250 ml of ice-cold saline, 4% paraformaldehyde was slowly perfused for about 30 min, until the right atrium flowed out clear paraformaldehyde and the lungs turned white. The lumbar spinal cord tissue was immediately removed into the 4 °C paraformaldehyde and fixed it for 24 h, then dipped it in clean water for 24 h and changed the water three times in the middle. At last, it embedded in paraffin. Continuous slice was conducted within 2 mm of lumbar-3 spinal cord tissue, with a thickness of 10 um.

### Tissue and serum MDA assay

In the spinal cord and serum, lipid peroxidation was determined as malondialdehyde (MDA) concentration. After SCII, MDA levels in the damaged spinal cord were measured based on reaction with thiobarbital acid using MDA assay kits (Nanjing Jiancheng Bioengineering Institute, Nanjing, China) per manufacturer’s instructions. Using N-methyl-2-phenylindole as substrate, intracellular MDA concentration was calculated by measuring maximal absorbance at 532 nm on a spectrophotometer. MDA concentrations were expressed as nanomoles per milligram of spinal cord protein (nmol/mg prot) in spinal cord homogenate and nanomoles per milliliter (nmol/ml) in serum.

### Tissue and SOD analysis

Serum superoxide dismutase (SOD) activity in serum and spinal cord homogenate was measured with xanthine oxidase assay kits (Nanjing Jiancheng, Bioengineering Institute, Nanjing, China) per manufacturer’s instructions. The principle of this method is based on inhibition of nitro-blue tetrazolium reduction by xanthine–xanthine oxidase system as a superoxide generator. SOD was calculated by measuring maximal absorbance at 550 nm on a spectrophotometer. One unit of SOD was defined as the enzyme amount causing 50% inhibition in NBT reduction. The SOD activity was expressed as units per milligram of spinal cord protein (U/mg prot) in spinal cord homogenate and nanomoles per milliliter (nmol/ml) in serum.

### H&E staining

Taking the spinal cord biopsies embedded in paraplast from specimen collection step. Representative 5 μm sections of paraffin-embedded tissue were cut and deparaffinized with xylene, graded ethanol, then mounted on slides for hematoxylin and eosin (H&E) staining. The damaged neurons were identified by the loss of Nissl substance, the cavitation around the nucleus, and the presence of pyknotic homogenous nuclei.

### Immunohistochemistry staining

The tissue sections were deparaffinized and blocked in 2% normal horse serum for 2 h and were then incubated with a primary survivin antibody at 4 °C overnight. After three washes in phosphate-buffered saline, the corresponding secondary antibody was added, followed by incubation for 2 h at room temperature. The sections were then rinsed and placed in the avidin-peroxidase conjugate solution for 2 h. Horseradish peroxidase was detected with 0.05% diaminobenzidine. After that, the sections were counterstained with hematoxylin, dehydrated, and mounted. Appropriate sections were used as positive and negative controls. Five microscopic fields of positive cells were chosen for imaging.

### Survivin western blot analysis

To confirm survivin expression, we performed a western blot. Procedures were all performed per manufacturer’s instructions (BCA Protein Assay Kit). Briefly, frozen homogenate tissues from specimen collection step were thawed. Protein lysate buffer was added to 20 mg of frozen tissues (with 2.0 μL protein lysate per 1.0 mg tissue). Each sample containing 20 μg total protein was loaded onto a 12% separating gel and transferred to membranes. After washing once with Tris-buffered saline with Tween 20(TBST) for 5 min and blocking in 5% skim milk in TBST for 4 h at room temperature, the membranes were incubated overnight with primary antibodies, anti- survivin (1:100, Santa Cruz, CA), and anti-β-actin (1:2000) at 4 °C. After washing, membranes were incubated with horseradish-peroxidase (HRP)-labeled goat anti-mouse (1:10000) secondary antibody (Jackson) for 4 h at room temperature. The color was developed using enhanced chemiluminescence, and images were analyzed with LabWorks gradation image analysis software. Each experiment was repeated thrice.

### Terminal-deoxynucleotidyl transferase-mediated nick end labeling assay

TUNEL assay was performed to detect apoptosis. In brief, the sections fixed by paraformaldehyde were quenched in 3% hydrogen peroxide for 10 min, washed with phosphate-buffered saline, and incubated with terminal deoxynucleotidyl transferase enzyme for 1 h at 37 °C. The reaction was stopped by washing in phosphate-buffered saline, and anti-digoxin-peroxidase was then added to the slides. After another wash, the sections were incubated with diaminobenzidine for 10 min at room temperature, followed by counter-staining with hematoxylin and dehydration. Distilled water, as a substitution for terminal deoxynucleotidyl transferase, was used as negative control. The number of TUNEL-positive cells in the five microscopic fields was counted.

### Statistical evaluation

All data were reported as mean ± standard deviation. All statistical analyses were conducted using statistical analysis software (SPSS, Version 18.0). In experiments involving histology or immunohistochemistry, all figures shown are representative of at least three experiments performed on different experimental days. For statistical evaluation, one-way analysis of variance (ANOVA) was employed. Independent samples *t* test was used to compare value of BBB score/SOD/MDA and the numbers of survivin, TUNEL-positive cells between the reperfusion groups and the GRb1 treatment groups. Pearson correlation analysis was also performed to some index. *P* value < 0.05 was considered statistically significant.

## Results

### Rats behavioral test results

The results (Fig. 1b) showed that the BBB score of rats after the ischemia-reperfusion injury is significantly decreased. Although the values of each subgroup in the drug group are lower than those in sham group, the values of each subgroup are higher than those in the ischemia-reperfusion group (*p* < 0.05). With the increase of the dosage of drug intervention, the score also increased, but the score of 80 mg/kg changed little compared with 40 mg/kg.

### Effects of GRb1 on oxidant stress after SCII

#### Determination of MDA content in serum and spinal cord tissues

As can be seen from Fig. 1d, e, the content of MDA in serum and spinal cord increased significantly after ischemia-reperfusion injury. Although the content of MDA of each subgroup in the drug group was higher than those in the sham group, the activity of each group was decreased compared with that in the ischemia-reperfusion group (*p* < 0.05). With the increase of drug intervention dose, the content of MDA decreases correspondingly, but the content of MDA changes little in 40 mg/kg compared with 80 mg/kg.

#### Determination of SOD activity in serum and spinal cord tissues

From Fig. 1f, g, it can be seen that the SOD activity of serum and spinal cord decreased significantly after ischemia-reperfusion injury. The SOD activity of each subgroup in the drug group was lower than that in the sham group, but the activity of each group was increased compared with that in the ischemia-reperfusion group (*p* < 0.05). The activity of SOD increased with the increase of drug intervention dose. However, the activity of SOD changed little in 40 mg/kg compared with 80 mg/kg.

### Effect of GRb1 on morphological changes after SCII

**Fig. 2:**
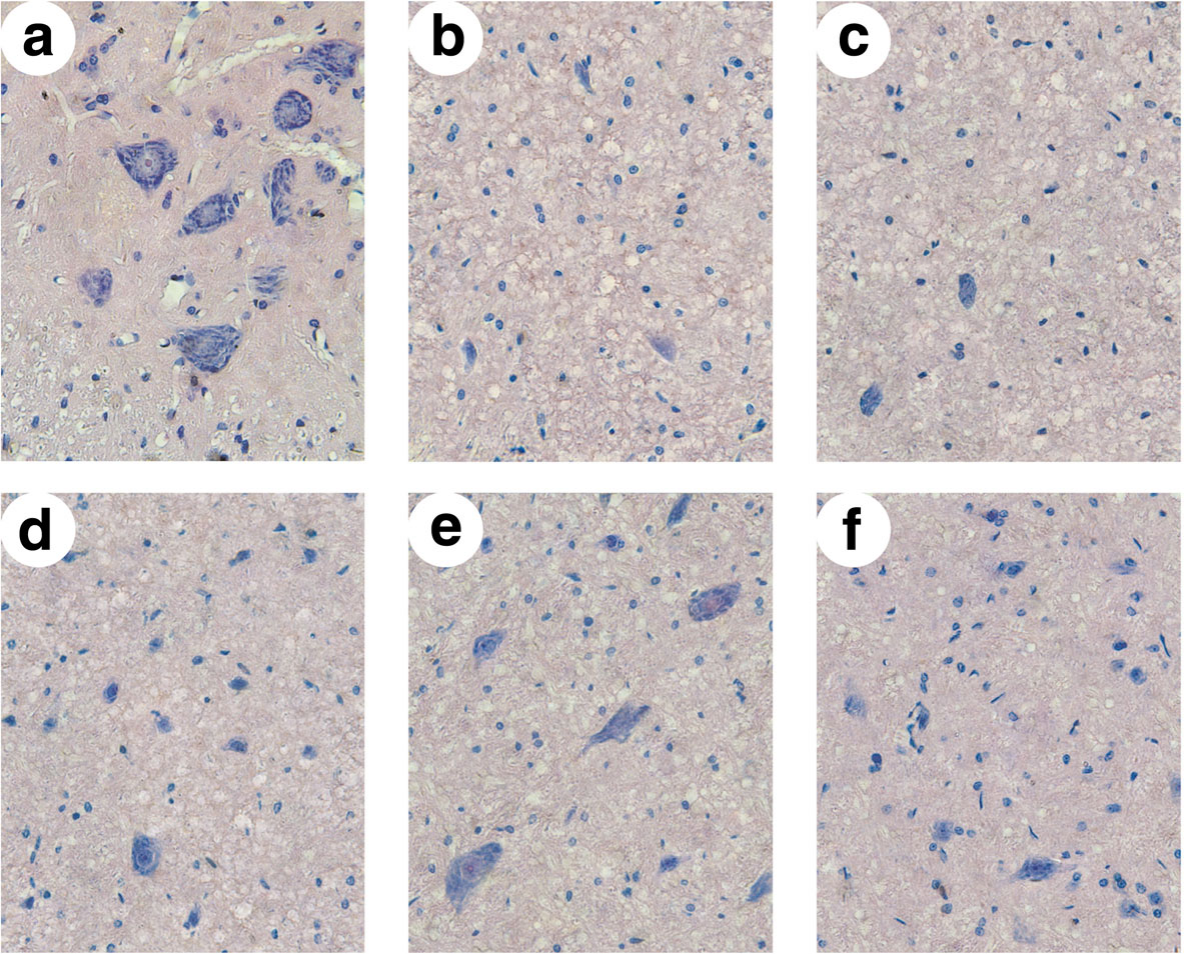
H&E staining of sections from different groups. **a** Sham group. **b** SCII model group. **c** GRb1 treatment groups 10 mg/kg. **d** GRb1 treatment groups 20 mg/kg. **e** GRb1 treatment groups 40 mg/kg. **f** GRb1 treatment groups 80 mg/kg. Normal appearing motor neurons were seen in the sham group, and GRb1 treatment attenuated the histopathologic damage to the spinal cord after reperfusion. Scale bar 20 μm

HE staining images showed that the Nissl bodies in the cytoplasm of normal spinal cord neurons were clearly visible (Fig. 2a). After ischemia-reperfusion, the neurons shrank, the Nissl bodies were blurred, and the number of Nissl bodies decreased (Fig. 2b). The spinal cord tissue showed vacuolar degeneration of different sizes. This kind of injury change was found in all sub-groups of the drug group, but compared with the ischemia-reperfusion group, the damage degree was obviously lighter (Fig. 2c–f).

### Effects of GRb1 on expression of survivin after SCII

**Fig. 3:**
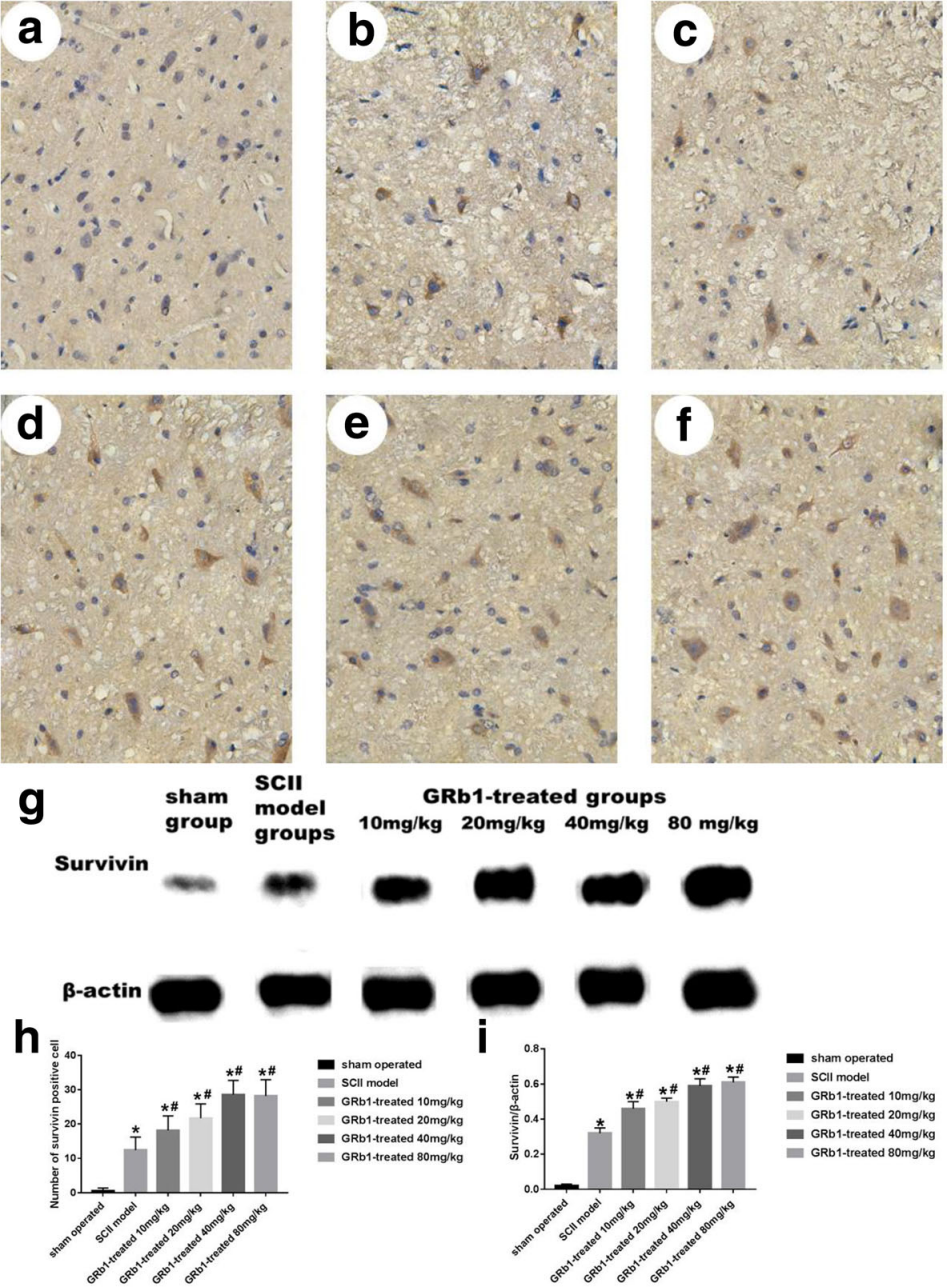
The expression of survivin in spinal cord tissue. Survivin staining of spinal sections from different groups. **a** Sham group. **b** SCII model group. **c** GRb1 treatment groups 10 mg/kg. **d** GRb1 treatment groups 20 mg/kg. **e** GRb1 treatment groups 40 mg/kg. **f** GRb1 treatment groups 80 mg/kg. **g** The expression of survivin was tested by western blot. **h** Quantification of survivin positive cell. **i** Comparison of densitometric quantitation of gel bands between sham, SCII model, and GRb1 treatment groups in **g**. The plasma and processes were stained brown in survivin-positive cells. The results showed that compared with the SCII model group, the values of survivin content in all subgroups of the drug group increased. Scale bar 20 μm. (*n* = 15 in each group, *P < 0.05, compared with the sham group; #*P* < 0.05, GRb1 vs. SCII group)

As can be seen from Fig. 3, there was a significant increase in survivin protein-positive cells in the anterior angle of the spinal cord in rats after ischemia-reperfusion injury (Fig. 3b). Compared with the ischemia-reperfusion group, the number of survivin positive cells in each subgroup of the drug group increased (Fig. 3c–f). With the increase of drug intervention dose, survivin protein-positive cells increase correspondingly. However, the number of survivin positive cells did not change much in 40 mg/kg compared with 80 mg/kg.

From Fig. 3g, we can see that survivin protein is not expressed in spinal cord neurons before ischemia, and it expressed immediately after ischemia-reperfusion. The expression level of survivin protein increased with the dose of drug intervention. Compared with the ischemia-reperfusion group, the expression level of survivin protein increased in the subgroup value of the drug group. However, compared with 80 mg/kg, the expression level of survivin protein 40 mg/kg did not change much.

### Effect of GRb1 on apoptosis after SCII

**Fig. 4:**
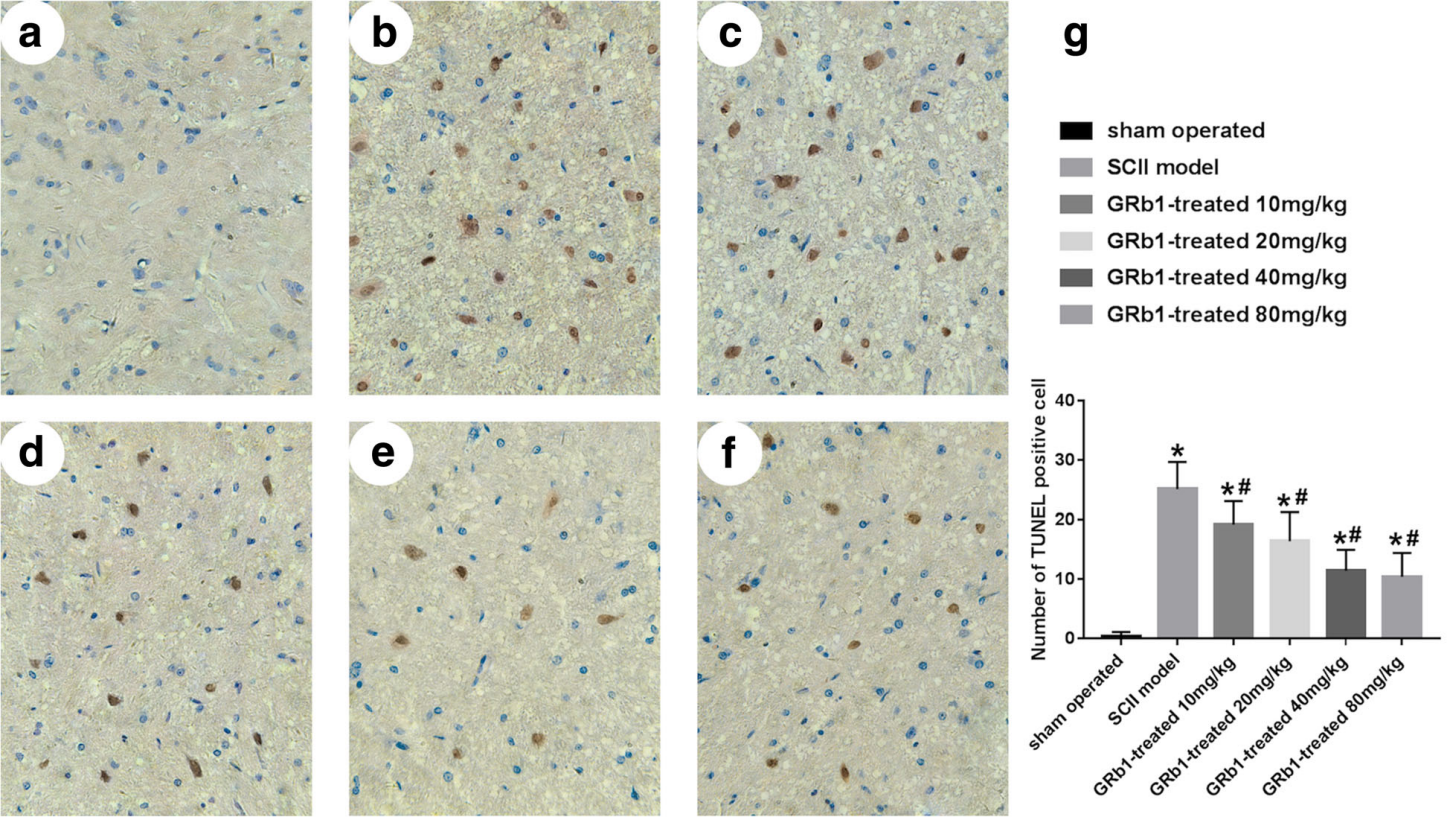
TUNEL staining of spinal sections from different groups. GRb1 treatment reduced the neuron apoptosis at different time points after reperfusion. **a** Sham group. **b** SCII model group. **c** GRb1 treatment groups 10 mg/kg. **d** GRb1 treatment groups 20 mg/kg. **e** GRb1 treatment groups 40 mg/kg. **f** GRb1 treatment groups 80 mg/kg. **g** Quantification of TUNEL positive cell. The plasma was stained brown in TUNEL-positive cells. Scale bar 20 μm. (*n* = 15 in each group, *P < 0.05, compared with the sham group; #*P* < 0.05, GRb1 vs. SCII group)

As can be seen from Fig. 4, there was also a significant increase in TUNEL-positive cells in the anterior angle of the spinal cord in rats after ischemia-reperfusion injury (Fig. 4b). The number of TUNEL-positive cells in each subgroup of the drug group was significantly lower than that in the ischemia-reperfusion group (*p* < 0.05). The number of TUNEL-positive cells decreased significantly with the increase of the drug intervention dose, but the number of TUNEL-positive cells did not change significantly when compared with 40 mg/kg and 80 mg/kg. The correlation coefficient of survivin-positive cells and TUNEL positive cells is − 0.601.

## Discussion

In this study, we established a model of spinal cord ischemia-reperfusion injury in rats. Through the intervention of GRb1, the detection of SOD, MDA, survivin protein expression, and apoptosis were used to show the partial protective mechanism of GRb1 for spinal cord ischemia-reperfusion injury.

Oxidative stress is an important mechanism in spinal cord ischemia-reperfusion [13]. Nitric oxide, superoxide anions, hydrogen peroxide, and hydroxyl radicals were produced during oxidative stress, which could cause damage to membranes and basic organelles by peroxidation of unsaturated fatty acids in membrane phospholipids, and also caused cell death through necrosis or apoptosis patterns [14]. Many reactive oxygen species (ROS) are produced at a low level under physiological conditions, but under oxidative stress conditions, especially when the generation of ROS exceeds the scavenging capacity of antioxidant enzymes such as SOD, it can lead to cell damage and neural tissue damage [15, 16]. SOD is an important antioxidant enzyme widely distributed in various organisms [17]. It is the primary substance for scavenging free radicals in organisms and is the preferred indicator for the antioxidant capacity of organisms. Therefore, many scholars used SOD activity as an intuitive indicator of the degree of oxidative stress damage in the ischemia-reperfusion injury. MDA can be formed by condensation of acetaldehyde and ethyl formate. In vivo, the end product of lipid peroxidation of free radicals is MDA, which can cause cross-linking of many biomacromolecules and is cytotoxic [18]. MDA is also an important monitoring index of oxidative stress damage during ischemia-reperfusion injury, which is widely used in such experiments. As can be seen from the results of this experiment, spinal cord ischemia-reperfusion injury can significantly reduce SOD activity in serum and spinal cord tissue of rats. But the content of MDA increased significantly. These results suggest that ischemia-reperfusion injury of the spinal cord in rats can induce obvious oxidative stress. Although the subgroup values of the drug group were lower than those of the sham group, the SOD activity was increased and the MDA content was decreased compared with the ischemia-reperfusion group. The change was more obvious with the increase of the intervention dose, and the trend was not obvious after the intervention dose was more than 40 mg/kg. The result confirms that GRb1 can increase SOD activity and reduce MDA production in rats, and this trend is no longer obvious after the intervention dose is more than 40 mg/kg.

Survivin is made up of 142 amino acids, which can function only by forming homodimers [19]. Survivin has only one baculovirus IAP repeats (BIR) domain, which is very important for the formation of dimer and the inhibition of apoptosis such as binding to caspases. The BIR domain at the N-end of the survivin protein can inhibit the activity of caspase-3 and caspase-7 to inhibit apoptosis. The c-terminal of survivin protein does not have the RING structure that other members of the IAP family have. And the survivin protein monomers can aggregate and bind to each other through the BIR domain to form a symmetrical dimer, which is necessary for the survivin protein to resist apoptosis. Survivin proteins are highly cell-selective and highly expressed in the tissues and organs of embryos and fetuses [20]. At present, most studies focus on tumors such as hepatocellular carcinoma and lung cancer [21–23]. Only a small number of studies on cerebral ischemia-reperfusion injury suggest that survivin protein can be expressed after brain ischemia-reperfusion injury [24, 25]. It can be seen from the results of this experiment that SCII can increase the number of survivin protein-positive cells in the anterior horn of spinal cord and increase the expression of survivin protein in spinal cord tissue, indicating that SCII can promote rat spinal cord neurons express survivin protein. Compared with the ischemia-reperfusion group, the number of survivin positive cells and the expression level of survivin protein increased in each subgroup of the drug group. The change became more obvious with the increase of the dose of the intervention drug, but the trend was not obvious after the intervention dose was more than 40 mg/kg. These results suggest that GRb1 intervention can promote the expression of survivin protein in rat spinal cord neurons, and this trend is not obvious after the intervention dose is more than 40 mg/kg. And this change has an inverse trend with the changes of apoptotic neurons in the anterior horn of rat spinal cord, and the correlation coefficient is − 0.601.

GRb1 has been shown to be a ligand for glucocorticoid receptors and androgen receptors, and they act as agonists to promote rapid ion influx and NO production [26, 27]. There is a preclinical systematic review to investigate the efficacy of GRb1 for animal models of myocardial ischemia/reperfusion injury. This study suggests that GRb1 is a potential cardio-protective candidate for further clinical trials of myocardial infarction. A clinical study showed that GRb1 has therapeutic effects on cardiac function and remodeling in patients with heart failure [28]. Analysis of GRb1 metabolites has been reported to detect 14 metabolites in rat urine, feces, stomach, and large intestine [29, 30]. After intravenous injection of drugs, urine mainly contains prototype drugs and some metabolites. The peak time for blood medicines was about 1.02 h [31]. The bioavailability by oral administration is low, and there are fewer prototype drugs entering the blood. On this basis, the metabolic reactions such as hydrolysis, binding, oxidation, and isomerization are also minimal. Most of the metabolites detected in urine after oral administration were metabolites of gastrointestinal flora, of which hydrolyzed products were the majority. And in urine, the amount of hydrolyzed metabolites was higher than that of the prototype drugs [32]. When GRb1 was given intravenously to healthy people, the peak concentration of GRb1 in plasma was 10.572 ± 8.925 mg/L and the drug peak time is 1.655 ± 0.144 h. The plasma terminal elimination half-life was 47.983 ± 7.256 h, so we speculated that the duration of the pretreatment treatment effect of GRb1 was about 2 days. After 150 h, the plasma concentration of the drug was still 0.889 ± 0.033 mg/L [33]. In this experiment, intraperitoneal injection of drugs is used to ensure the blood concentration on the one hand and sufficient drug metabolism in the liver on the other hand. Some people used 1200 mg/kg oral drug concentration to carry out the test, and the results showed that the linear relationship between the blood concentration in the range of 1–20.0 mg/L was good, and then the rising trend slowed down. Considering that the bioavailability of the drugs absorbed by abdominal cavity is higher than that by oral administration, but there is still some loss, so the 20 mg/kg group in the experimental group has not achieved the best effect. After deducting the fixed loss, the upper limit of blood concentration may have been reached in the 40 mg/kg group. Similarly, the 80 mg/kg group also reached the upper limit. So there is little difference between the 40 mg/kg group and the 80 mg/kg group in all aspects of evaluation effect. It can be seen from this experiment that GRb1 can play a protective role in the process of SCII by antioxidant, promoting survivin protein expression and inhibiting apoptosis. Moreover, the protective effect increases with the increase of the dose within the range of 10–40 mg/kg, but no longer increases after the dose exceeds 40 mg/kg. It suggested that the effective dose is within the range of 10–40 mg/kg. This provides an animal experimental basis for the clinical application of GRb1.

## Conclusion

Preconditioning of GRb1 could protect rat spinal cord from ischemia-reperfusion injury through anti-oxidation, promoting survivin protein expression and inhibiting apoptosis. The protective effect of the intervention dose within the range of 10–40 mg/kg was enhanced with the increase of the dose, while the protective effect was no longer enhanced after the dose exceeded 40 mg/kg.

## Data Availability

The datasets used and/or analyzed during the current study are available from the corresponding author on reasonable request.

## Abbreviations

BBB: Basso, Beattie, and Bresnahan
GRb1: Ginsenoside Rb1
H&E: Hematoxylin and eosin
i.p.: Intraperitoneal injection
MDA: Malondialdehyde
SCII: Spinal cord ischemia-reperfusion
SD: Sprague-Dawley
SOD: Superoxide dismutase
TUNEL: Terminal dUTP nick-end labeling
